# Nasopharyngeal carcinoma in Ibadan, Nigeria: a clinicopathologic study

**DOI:** 10.11604/pamj.2020.36.82.19657

**Published:** 2020-06-09

**Authors:** Gabriel Olabiyi Ogun, Aralola Adepeju Olusanya, Victor Ifeolu Akinmoladun, Adebolajo Adewunmi Adeyemo, Segun Ayodeji Ogunkeyede, Adekunle Daniel, Babatope Lanre Awosusi, Ebenezer Oluwaseun Fatunla, Ayotunde James Fasunla, Paul Adekunle Onakoya, Aderemi Adeleke Adeosun, Onyekwere George Nwaorgu

**Affiliations:** 1 Department of Pathology, College of Medicine, University of Ibadan, Ibadan, Nigeria,; 2 Department of Pathology, University College Hospital, Ibadan, Nigeria,; 3 Department of Oral and Maxillofacial Surgery, College of Medicine, University of Ibadan, Ibadan, Nigeria,; 4 Institute of Child Health, College of Medicine, University of Ibadan, Ibadan, Nigeria,; 5 Department of Otorhinolaryngology, University College Hospital, Ibadan, Nigeria,; 6 Department of Otorhinolaryngology, College of Medicine, University of Ibadan, Ibadan, Nigeria

**Keywords:** Nasopharyngeal carcinoma, Ibadan, Nigeria, clinical features, histopathology, nasopharynx, carcinoma

## Abstract

**Introduction:**

nasopharyngeal carcinoma is relatively common in our environment. It is one of the most difficult malignancies to diagnose at an early stage. The aim of the study was to determine the clinical features, clinical disease stage of nasopharyngeal carcinoma at presentation and at diagnosis as well as the histologic types at the University College Hospital, Ibadan, Nigeria.

**Methods:**

this was a ten year retrospective study of all histologically confirmed nasopharyngeal carcinoma between January 2007 to December 2016 using clinical and pathology records and files.

**Results:**

there were 73 cases. The male: female ratio was 1.7. The age of patients ranged from 12 to 80 years with a mean age of 39 ± 16 years. The median age at diagnosis was 40 years. The peak age group of occurrence was 40-49 years. The most common symptoms were namely epistaxis in 67.1% of patients at presentation, neck mass/swelling (64.4%) and nasal mass/obstruction (63.0%). Majority (54.8%) of the patients presented late with stage 3 or 4 disease. Most (94.5%) of the tumours were of the non-keratinizing squamous cell carcinoma subtype. The keratinizing and basaloid variants accounted for 4.1% and 1.4% of the tumours respectively.

**Conclusion:**

vague, non-specific symptoms make patients present at late stages of the disease, making it almost impossible to attempt cure. The dominant histopathological type is non-keratinizing squamous cell carcinoma and resembles that seen in most parts of Nigeria and endemic areas of the world.

## Introduction

Nasopharyngeal carcinoma (NPC) is defined by the World Health Organization (WHO) as carcinoma arising in the nasopharyngeal mucosa showing light microscopy and ultrastructural evidence of squamous differentiation [1]. Based on the current WHO classification of tumours of the nasopharynx, the three histomorphologic types are namely keratinizing, non-keratinizing (differentiated and undifferentiated; formerly WHO Grade II and III respectively) and basaloid squamous cell carcinoma [1]. It is a common cancer and leading cause of morbidity and mortality in well-defined populations of the world especially in natives of Southern China, Southeast Asia, the Arctic and the Middle East/North Africa [2]. The distinctive racial/ethnic and geographic distribution of NPC worldwide suggests that both environmental factors and genetic traits contribute to its development [2]. The Globocan statistics on cancer incidence worldwide for 2018 gave an age standardized incidence and mortality of 1.5 and 0.84 per 100,000 respectively for NPC, with a 5 year prevalence rate of 4.4 per 100,000 [3-5]. In NPC endemic countries and regions of the world such as Southern China, Hong Kong and Greenland, incidence rates are as high as 25-50 per 100,000 [3-5].

Worldwide incidence of NPC is 2-3 times commoner in men [1,2,6]. Generally in the endemic regions of the world the disease has a peak incidence in the 50-59 age group and then decline, in contrast to low risk population where there is a gradual increase in the incidence rate with increasing age [1,2,6]. Clinically, NPC typically run a long course of asymptomatic disease and usually the effect of loco regional disease like metastasis to cervical lymph nodes or cranial palsies are the first presentation of the disease, hence patients tend to present for the first time with advanced disease and poor prognosis [1,2,7-13]. The origin of NPC, its presentation, histopathological types, treatment and prognosis differ from those of other malignant neoplasm originating from the upper aerodigestive tract [8]. The majority of publications from Nigeria lump NPC with other head and neck malignancies hence making it difficult to find out the true characteristics of this tumour locally. This study aims to determine the clinical features and clinical disease stage of nasopharyngeal carcinoma at presentation and diagnosis as well as the histologic types at the University College Hospital, Ibadan, Nigeria.

## Methods

**Study design and patient population:** all cases of nasopharyngeal carcinoma included in this study were diagnosed histologically, over the study period (January 2007 to December 2016), as available from the records and files of the department of pathology, University College Hospital, (UCH), Ibadan, Nigeria, a referral hospital that receives cases of head and neck cancers from across mainly Southwest Nigeria. The cases were retrieved, reviewed, diagnosis confirmed and histologic type was ascribed using the 2005 and 2017 WHO classification of NPC (which are namely keratinizing squamous cell carcinoma; non-keratinizing squamous cell carcinoma and basaloid squamous cell carcinoma). The clinical files and notes of the patients were also reviewed. Subsequently demographic data, detailed clinical presentation and clinical stage at presentation were retrieved. The staging system used in this study is the TNM classification of carcinomas of the nasopharynx, seventh edition, 2009 [14].

**Statistical analysis:** all the data retrieved were entered into Microsoft Excel and subsequently into Statistical Package for Social Sciences version 22 (IBM Corp. Armonk, NY) for statistical analysis. Descriptive statistics was performed on the data generated and presented as frequencies, mean and ranges.

**Ethical issues:** ethical clearance for the study was obtained from the joint University of Ibadan/University College Hospital, Ibadan, Nigeria ethical review committee (UI/EC/18/0163). Furthermore, this study was conducted in compliance with the guidelines of the Helsinki declaration on biomedical research in human subjects. Confidentiality of the identity of the patients and personal health information was maintained.

## Results

**Figure 1:**
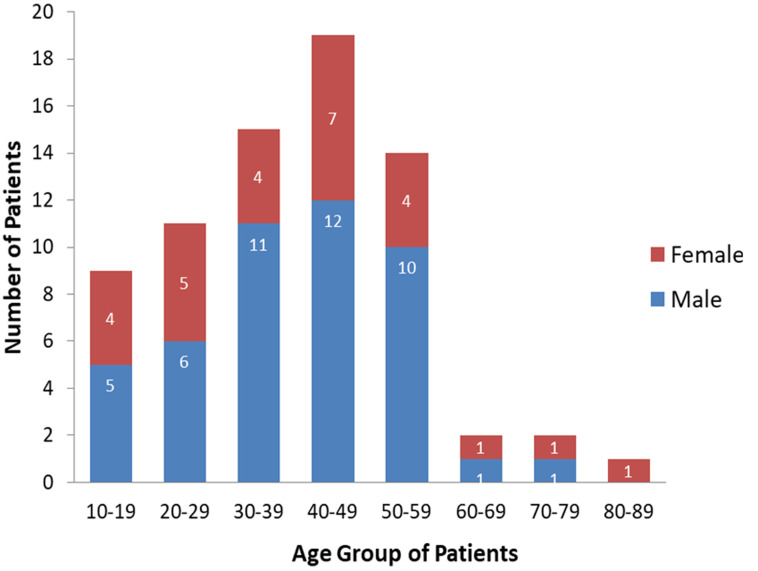
age groups and gender distribution of the patients

**Figure 2:**
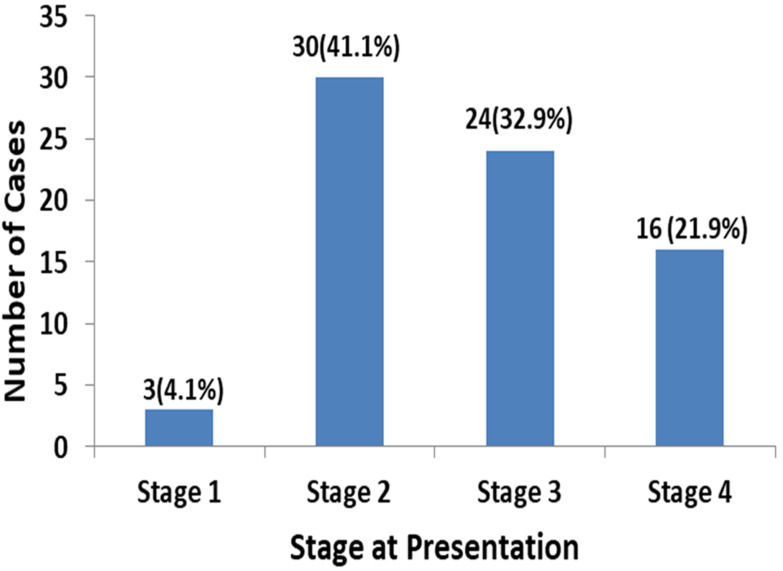
clinical stage at presentation showing percentages for each stage

**Figure 3:**
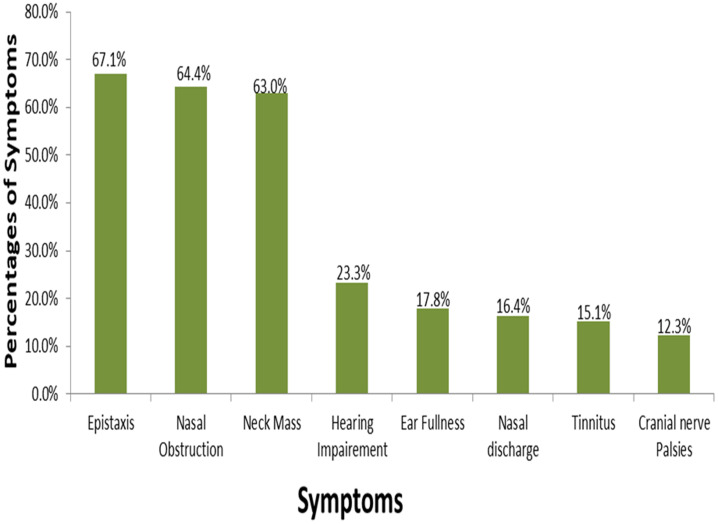
relative ratio and percentages of the most common symptoms at first presentation

A total of 73 cases of nasopharyngeal carcinoma were diagnosed in the University College Hospital, Ibadan, Southwest Nigeria during the period under review. There were forty-six (63.0%) males and 27 were females, male: female ratio is 1.7. The age of patients ranged from 12 to 80 years with a mean age of 39 ± 16 years. The median age at diagnosis was 40 years. Figure 1 shows that the peak age group of occurrence is 40-49 years and it account for 26.0% of the patients, 30-39 years (20.5%) and 50-59 years (19.25%). More than 90% of the patients were aged less than 60 years. Clinically, majority (54.8%) of the patients presented late with stage 3 or 4 disease (Figure 2) with only 3 (4.1%) presenting with stage 1 disease. Table 1 shows all the presenting symptoms in patients, while Figure 3 shows the most common symptoms; namely epistaxis in 67.1% of patients at presentation, neck mass/swelling (64.4%) and nasal mass/obstruction (63.0%). A proportion of the patients also had ear symptoms including hearing impairment/loss (23.3%), ear fullness (17.8%) ear discharge (16.4%) and tinnitus (15.1%). Nine (12.3%) patients had cranial nerve palsies at presentation. Histologically, most (94.5%) of the tumours were of the non-keratinizing squamous cell carcinoma subtype. The keratinizing and basaloid variants accounted for 4.1% and 1.4% of the tumours respectively.

**Table 1:** percentages of all different clinical symptoms in patients at first presentation

CLINICAL SYMPTOMS	FREQUENCY (%)
**Nasal symptoms**	
Epistaxis	49 (67.1)
Nasal obstruction	46 (63.0)
Nasal discharge	12 (16.4)
**Neck mass/Lymph node enlargement**	47 (64.4)
**Ear symptoms**	
Hearing impairment	17 (23.3)
Fullness	13 (17.8)
Tinnitus	11 (15.1)
Otalgia	2 (2.7)
Vertigo	1 (1.4)
**Neuro-Ophthalmic**	
Cranial nerve palsies (mainly 5^th^ and 6^th^ CN)	9 (12.3)
Hemifacial dysaesthesia	1(1.4)
Anosmia	1(1.4)
**Other masses**	
Nasopharyngeal	6 (8.2)
Palatal	2 (2.7)
Oral	1 (1.4)
Oropharyngeal	1 (1.4)

## Discussion

The male to female ratio in this study was 1.7:1. This is in keeping with studies from different parts of the world including Nigeria that shows that the disease predominantly affects men in both endemic and non-endemic populations with ratio as high as 3:1 [1,2,6,8-10,13,15-28]. Typically, NPC is a disease that occurs predominantly in adults with peak occurrence in the 4^th^ to 6^th^ decade of life. Our observation from this study was that there was a gradual increase in the incidence from the second decade of life, with a peak in the 40-49 years (Figure 1) with subsequent precipitous drop in incidence after the age group 50-59 years. This pattern of age group distribution is typically observed in non-endemic population in contrast to endemic population where there are two age group peaks, usually a minor peak in the young and a more prominent peak in much older age groups, usually in the 6^th^ decade of life [2,6,15]. Typically, NPC presents in the early phase of the disease with nonspecific symptoms leading to delay in patients´ seeking medical attention and where there is visit to the hospital, the symptoms are treated as benign condition as reported from different studies from all parts of the world [6,8,11-13,20,23,25,27-29]. The delay in diagnosis therefore makes the attempt at curative therapy very difficult. All the 73 patients reviewed in this study presented with symptoms that are well established in literature with regards to NPC.

Clinical presentation of nasopharyngeal carcinoma depends on the extent of the disease at first contact with a physician. The progression of direct tumour invasion anteriorly is usually into the nasal cavity, pterygoid fossa and maxillary sinuses; laterally the tumour goes beyond the pharyngobasilar fascia into the parapharyngeal and infratemporal spaces; and at the base of the skull, clivus and into the intracranial space and structures when the disease extends posteriorly and superiorly [30]. Therefore, the anatomic spread will determine the stage at presentation. As illustrated in a review by Petersson [6], the presenting symptoms of patients with NPC can be divided into 4 groups: related to a mass in the nasopharynx (epistaxis, obstruction and discharge); related to eustachian tube dysfunction (decreased hearing, tinnitus); skull base involvement (erosion) with impairment of the 5^th^ and 6^th^ cranial nerves (headache, diplopia, facial pain and numbness/paresthesia); and neck mass. In a large retrospective study of 5020 patients with NPC by Lee *et al*. from Hong Kong, the initial presenting symptoms were, in descending frequency, (the top 5 symptoms): neck mass (75.8%), nasal discharge, bleeding, obstruction (73.4%), aural tinnitus, impairment of hearing (62.4%), headache (34.8%), ophthalmic diplopia, squint (10.7%) [15]. The most common presenting features from this study were epistaxis in 67.1% of patients, neck mass in 64.4% and nasal obstruction (63.0%), hence our findings show frequency of symptoms similar to that by Lee *et al*.

In various studies, the most common symptoms at presentation are neck mass and nasal symptoms which include nasal bleeding and obstruction but the frequency of such presentation varies in different studies. However, the frequency of neck mass in this study is comparable with that observed by Al-Rajhi *et al*. in Saudi Arabia who recorded neck mass frequency of 61.6% [31]. Adewuyi *et al*. and Iseh *et al*. working in Northern Nigeria found neck mass in more than 90% of patients and nasal obstruction in up to 75% at first presentation [23,28]. It can be inferred that more patients in the two studies presented much later than our study despite the fact that all the studies were done in Nigeria. There is significant difference in the geographic location, with relative poorer socio-economic status which may make later presentation the rule, in Northern Nigeria compared to Southwest Nigeria where our study was done. About 12.3% of patients in our study presented with cranial nerve (CN) involvement which typically indicates skull base infiltration and erosion. An earlier study from our centre by Ogunleye *et al*. observed 25% of patients presented with various neuro-ophthalmic manifestation with cranial nerve involvement in only 18% of patients [20].

The frequency of CN palsies in our study is similar to that by Indudharan *et al*. from Malaysia but a higher frequency of 60% and 33.9% was recorded by Adewuyi *et al*. from Nigeria and Suzina *et al*. in another Malaysian study, respectively [9,28,32]. In general, the frequency of aural symptoms at presentation in this study (tinnitus- 15% and hearing impairment- 23.3%), is comparable to those observed by Indudharan *et al*. in their study, tinnitus and hearing impairment occurred in 12.3% and 18% of patients respectively [9]. Based on histological sub types, nonkeratinizing nasopharyngeal carcinoma (NK-NPC) showed the highest frequency of 94.5% in this study similar to figures of 94.6% in a recent study by Yates *et al*. from Zaria, Nigeria [22]. Studies from Nigeria, a non-endemic region, has shown that NK-NPC consistently have frequency above 85% in different cohorts [19,21,22,24,28]. An inference can be drawn that probably majority of NPC from Nigeria are Epstein Barr Virus (EBV) driven just like in endemic parts of the world and this is somewhat suggested by studies of EBV latent membrane protein (LMP)-1 detection by immunohistochemistry (IHC) in Nigerian studies by Omosebi *et al*. and Yates *et al*. [33,34].

The proportion of keratinizing NPC recorded in this study, in a non-endemic area, is very low (4%). Typically NK-NPC predominates in endemic regions, therefore, our finding of predominantly NK-NPC in a non-endemic region reinforces the notion that EBV plays a significant role in the development of NPC in this part of the world [2,6,12,30,35]. Only a single basaloid NPC variant was recorded in this study just like Yates *et al*. further supporting the observation that this is an uncommon histologic variant of NPC [1,6,22]. In this study, 54.8% of patients presented with locoregional disease and late clinical stage i.e. stages III and IV disease. This is similar to the 58.2% of patients in stage III and IV from a Ghanaian study and 61% by Perez *et al*. from an American study [29,36]. However, studies from Northern Nigeria show that stage III and IV tumour account close to 100% of cases [23,28]. Our study show significantly lower values for stage III and IV diseases when compared to studies from endemic areas where stage III and IV tumour account for close to 80-90% of cases at presentation [8,11,15,31]. The reason from the differences in percentage of patients presenting at different stages cannot be easily distilled out from these studies but can only be ascribed to a more aggressive course of disease prior to presentation. Tumour burden as reflected by the TNM stage is the most significant factor in terms of prognosis in NPC [1,2,6,7,35].

## Conclusion

Nasopharyngeal carcinoma in our locale is not very rare. The diseases occur predominantly in males with peak age incidence in the 40-49 years of age in both genders. The vague, non-specific symptoms make patients present at late stages of the disease, making it almost impossible to attempt cure. The dominant histopathological type NK-NPC, resembles that seen in most parts of Nigeria and endemic areas of the world, suggesting a role of EBV as a causal factor in NPC in Nigeria. It is advocated that patients with prolonged unexplained nasal symptom should have a thorough head and neck examination.

### What is known about this topic

- It is a leading cancer and leading cause of morbidity and mortality in well-defined populations of the world especially in natives of Southern China, Southeast Asia, the Arctic and the Middle East/North Africa;
- Typically run a long course of asymptomatic disease;
- Three histomorphologic types by the WHO are namely keratinizing, non-keratinizing and basaloid.

### What this study adds

- Histomorphologic pattern in a non-endemic area is similar to that of an endemic area;
- Majority of patients in our cohort presented with epistasis;
- Just about half of patients in our cohort presented with stage III and IV disease.
